# High Ambient Temperatures and Risk of Motor Vehicle Crashes in Catalonia, Spain (2000–2011): A Time-Series Analysis

**DOI:** 10.1289/ehp.1409223

**Published:** 2015-06-05

**Authors:** Xavier Basagaña, Juan Pablo Escalera-Antezana, Payam Dadvand, Òscar Llatje, Jose Barrera-Gómez, Jordi Cunillera, Mercedes Medina-Ramón, Katherine Pérez

**Affiliations:** 1 Centre for Research in Environmental Epidemiology (CREAL), Barcelona, Spain; 2 Universitat Pompeu Fabra (UPF), Barcelona, Spain; 3 CIBER Epidemiología y Salud Pública (CIBERESP), Barcelona, Spain; 4 Catalan Traffic Authority, Barcelona, Spain; 5 Servei Meteorològic de Catalunya, Barcelona, Spain; 6 Agència de Salut Pública de Barcelona, Barcelona, Spain; 7 Biomedical Research Institute Sant Pau (IIB Sant Pau), Barcelona, Spain

## Abstract

**Background:**

Experimental studies have shown a decrease in driving performance at high temperatures. The epidemiological evidence for the relationship between heat and motor vehicle crashes is not consistent.

**Objectives:**

We estimated the impact of high ambient temperatures on the daily number of motor vehicle crashes and, in particular, on crashes involving driver performance factors (namely distractions, driver error, fatigue, or sleepiness).

**Methods:**

We performed a time-series analysis linking daily counts of motor vehicle crashes and daily temperature or occurrence of heat waves while controlling for temporal trends. All motor vehicle crashes with victims that occurred during the warm period of the years 2000–2011 in Catalonia (Spain) were included. Temperature data were obtained from 66 weather stations covering the region. Poisson regression models adjusted for precipitation, day of the week, month, year, and holiday periods were fitted to quantify the associations.

**Results:**

The study included 118,489 motor vehicle crashes (an average of 64.1 per day). The estimated risk of crashes significantly increased by 2.9% [95% confidence interval (CI): 0.7%, 5.1%] during heat wave days, and this association was stronger (7.7%, 95% CI: 1.2%, 14.6%) when restricted to crashes with driver performance–associated factors. The estimated risk of crashes with driver performance factors significantly increased by 1.1% (95% CI: 0.1%, 2.1%) for each 1°C increase in maximum temperature.

**Conclusions:**

Motor vehicle crashes involving driver performance–associated factors were increased in association with heat waves and increasing temperature. These findings are relevant for designing preventive plans in a context of global warming.

**Citation:**

Basagaña X, Escalera-Antezana JP, Dadvand P, Llatje Ò, Barrera-Gómez J, Cunillera J, Medina-Ramón M, Pérez K. 2015. High ambient temperatures and risk of motor vehicle crashes in Catalonia, Spain (2000–2011): a time-series analysis. Environ Health Perspect 123:1309–1316; http://dx.doi.org/10.1289/ehp.1409223

## Introduction

Motor vehicle crashes are an important cause of mortality and disability worldwide (Lozano et al. 2012; World Health Organization 2013). Meteorological factors such as rain, snow, fog, wind, hail, and freezing temperatures are known to increase the risk of occurrence of motor vehicle crashes (Andrey et al. 2003). The effect of heat on the risk of motor vehicle crashes, however, has received less attention. High temperatures are known to decrease human capability of performing physical and intellectual tasks (Confalonieri et al. 2007), which in turn can increase the risk of traffic crashes. A number of experimental studies have consistently documented the negative effects of heat on driving performance (Daanen et al. 2003; Mackie and O’Hanlon 1977; Walker et al. 2001; Wyon et al. 1996), but few epidemiological studies have investigated this association at the population level.

Most of the existing epidemiological studies have examined the relationship between temperature and crashes using temporal aggregations (Antoniou et al. 2013; Bergel-Hayat et al. 2013; Coutin-Marie and Torres-Vidal 2010; Malyshkina et al. 2009; Nofal and Saeed 1997; Scott 1986). Some researchers have been able to perform analyses at the national level using monthly data. In France and the Netherlands, Bergel-Hayat et al. (2013) found that a 1°C increase in the monthly average temperature was associated with a between 1% and 2% increase in the number of crashes during the same month. In the state of Indiana (USA), an analysis of weekly averages found that weeks that were classified as high-risk in terms of crashes tended to have higher summer temperatures (Malyshkina et al. 2009). In Saudi Arabia, where temperatures above 40°C are common in summer, Nofal and Saeed (1997) found that the monthly number of crashes was correlated with increased monthly temperatures. However, a previous study of precipitation and traffic crashes reported a significant positive association based on daily data, in contrast with a significant negative association based on monthly data that the author attributed to lagged effects (Eisenberg 2004). Consequently, findings from studies using temporally aggregated data should be interpreted with caution.

Only a few epidemiological studies have used daily data. Among studies using daily time series, one found that more vehicle crashes were expected when temperatures were higher than the monthly mean (Brijs et al. 2008), another found a nonlinear relationship indicating more crashes at higher temperatures (Bergel-Hayat et al. 2013), and another found no relationship (Rosselló and Saenz-de-Miera 2011).

In a recent study of associations between extremely hot days and cause-specific mortality, we found a positive association with mortality resulting from traffic crashes (Basagaña et al. 2011). In the present study, we aimed to expand our previous findings by evaluating the association between high temperatures and motor vehicle crashes using a separate and more comprehensive data set including all motor vehicle crashes (not necessarily fatal crashes). Furthermore, we tested our hypothesized mechanism by evaluating the associations for crashes involving driver performance.

## Methods

*Design*. We performed a time-series analysis where the daily number of motor vehicle crashes was linked to daily temperatures while controlling for temporal trends. The study included all motor vehicle crashes resulting in human injuries or deaths that occurred during the warm period of the years 2000–2011 in the autonomous community of Catalonia (Spain). Catalonia has an area of approximately 32,000 km^2^ and had a population of 7.1 million in 2006 [Statistical Institute of Catalonia, IDESCAT; http://www.idescat.cat/ (in Catalan)]. The warm period was defined as the period between 15 May and 15 October, as these are the half-months with average maximum temperatures greater than 20°C (Basagaña et al. 2011).

*Motor vehicle crash data*. Data on individual motor vehicle crashes were obtained from the Catalan Traffic Authority [http://transit.gencat.cat/ca/ (in Catalan)] and included information on the date of the crash, the location where it occurred, the number of victims, the number of vehicles involved, the type of vehicles involved, and a set of concurrent factors assigned to the crash by the traffic authorities (e.g., positive alcohol test, traffic violation, bad weather, bad road conditions, distraction). We defined crashes with driver performance–associated factors as those that included among the list of concurrent factors at least one of the following: “Distraction,” “Driver error,” or “Disease, fatigue, or sleepiness.” Changes in the reporting of road traffic crashes were introduced starting in the period 2005–2007, which led to increases in the number of registered crashes. These changes involved the introduction of a new, more flexible and easy-to-use data entry system aimed at improving the underreporting of crashes of low severity.

**Figure 1:**
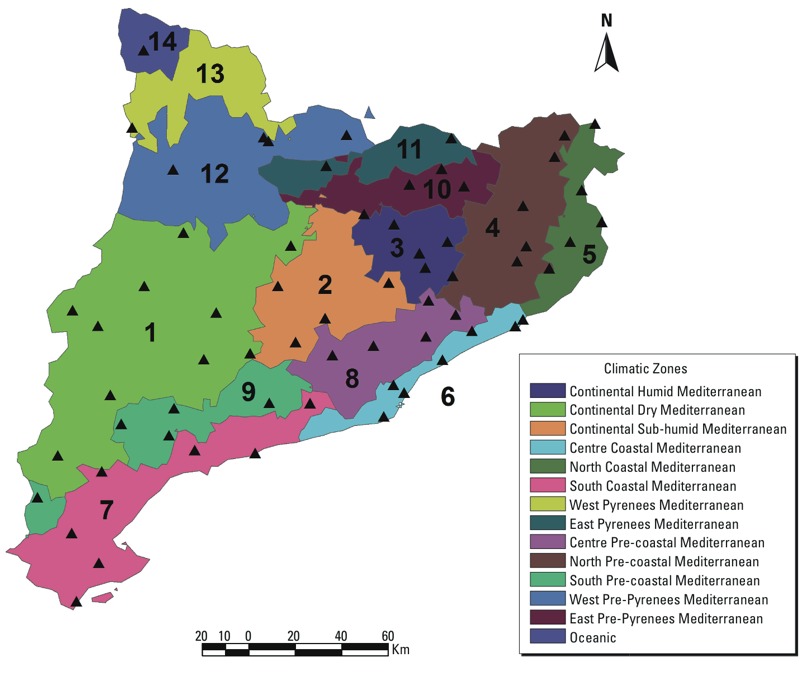
Map of climatic regions (identified by numbers) and weather stations (triangles).

*Meteorological data*. Daily maximum and minimum temperature and precipitation data were obtained from the Spanish Meteorological Association [AEMET; http://www.aemet.es/ (in Spanish)] and the Catalan Service of Meteorology [METEOCAT; http://www.meteo.cat/ (in Catalan)]. We obtained data from 66 weather stations covering Catalonia, with at least one station in each of the 14 different climate regions defined by the meteorological service according to temperature and precipitation patterns (Figure 1). There is no standard definition of heat waves, and the definitions used mostly depend on the purpose of the study (Smith et al. 2013). When looking at health effects, relative measures based on exceeding a certain temperature percentile during a number of consecutive days tend to produce stronger associations than measures based on absolute temperature values (Kent et al. 2014). Here, heat wave days were defined as days belonging to a period with ≥ 2 consecutive days exceeding the weather station–specific historic 95th percentile of maximum temperature, as the 95th percentile has been shown to be a good choice to capture mortality effects in our study area (Tobías et al. 2010). The choice of 2 consecutive days instead of a higher number is common (D’Ippoliti et al. 2010; Kent et al. 2014) and was made to avoid having low numbers in the statistical analyses.

*Exposure assessment*. Motor vehicle crashes were assigned the minimum and maximum temperature and precipitation values recorded on the date of the crash at the closest weather station within the same climatic region. Then, within each climatic region, traffic crashes were aggregated by date to obtain the daily number of motor vehicle crashes and the daily number of vehicle crashes with driver performance–associated factors. In the same process, temperature values from a given day were averaged by climatic region. A day in a specific climatic region was considered a heat wave day if at least one station in the region registered a heat wave during that day. For precipitation, a specific day in a climatic region was considered as having received some rain if at least one station in the region registered precipitation values > 0 mm.

*Statistical analysis*. The statistical analysis was performed in two steps. First, for each climatic region, we fitted Poisson regression models to separately relate the daily number of motor vehicle crashes to the maximum and minimum temperatures. The models were adjusted for precipitation (yes/no), day of the week, holidays (71 days in the entire period), days at the beginning or end of a holiday period (105 days in the entire period), and a strata variable uniquely identifying all combinations of year and month. These adjustments attempted to control for temporal trends and for differences in traffic volume, and they have been shown to produce consistent results when regional daily data on traffic volume were not available (Brijs et al. 2008; Rosselló and Saenz-de-Miera 2011). In addition, with these adjustments, comparisons were restricted to days within the same month of the same year; therefore, only short-term variations in temperature were assessed. Poisson regression models were used to estimate the relative risk (RR; obtained by exponentiating the regression coefficient) of motor vehicle crashes for each 1°C increase in the maximum or minimum temperature. In the second step, climatic region-specific RRs were combined using random effects meta-analysis to obtain the overall RR (Borenstein et al. 2010). The results were reported as percent differences [100 × (RR – 1)%]. The amount of heterogeneity by climatic regions was quantified using the *I*^2^ statistic (Higgins and Thompson 2002), which represents the proportion of total variation in effect estimates due to between-region heterogeneity.

When analyzing the daily number of vehicle crashes with driver performance–associated factors, the models were further adjusted for the remaining number of crashes that occurred during that day (i.e., crashes without driver performance–associated factors). This adjustment was expected to provide a better control for traffic volume of the day, as the resulting RR of temperature could be interpreted as the increase in risk of crashes with driver performance–associated factors for each 1°C increase in temperature on days that had the same number of vehicle crashes without driver performance–associated factors. The remaining number of vehicle crashes was included in the model with log-transformation after adding one unit (to prevent taking the logarithm of 0). This transformation was chosen to fulfill the linearity assumption (data not shown).

Analyses were further stratified by 4-year periods to assess temporal trends. Several sensitivity analyses were performed, modifying the original models by excluding crashes with alcohol or drugs as concurrent factors; excluding days with rain; excluding holidays, Sundays and the month of August (the month in which most people in Spain take vacations); adjusting for dummy variables resulting from the combination of year, month, and day of the week, resulting in a time-stratified case-crossover analysis (Levy et al. 2001); defining crashes with driver performance–associated factors as those that included “distraction,” “driver error,” or “disease, fatigue, or sleepiness” as the only concurrent factors; and repeating the analyses for driver performance–associated factors without adjusting for the number of motor vehicle crashes with factors unrelated to driver performance. The linearity of the associations for continuous variables was assessed using generalized additive models (GAM) (Wood 2006). The autocorrelation of the residuals was visually inspected using correlograms (Bhaskaran et al. 2013). Statistical significance was considered at the 5% level.

## Results

The present study included a total of 118,489 vehicle crashes with victims (dead or injured) that occurred between 15 May and 15 October of each year from 2000–2011, corresponding to an average of 64.1 crashes per day. A decrease in the number of crashes was observed from 2000 to 2004; however, this number increased in the period 2005–2007, mainly reflecting changes in the reporting of motor vehicle crashes, and decreased again at the end of the study period (Table 1). Overall, a driver performance–associated factor was reported for 32% of all crashes, although this percentage stabilized at approximately 40% in the last 4 years of the study period. Motor vehicle crashes were less common in August and on Sundays and were more common in July and on Fridays. A notable reduction in the number of crashes was observed during holidays. The distribution of motor vehicle crashes over regions mimicked that of the number of inhabitants, with the highest percentage of crashes occurring in the most populated regions (Table 2). Eighty-one percent of all crashes occurred in zones 5–8, which were located on or near the coast (Figure 1).

**Table 1:** Descriptive statistics on the daily number of motor vehicle crashes occurring between 15 May and 15 October of each year from 2000–2011.

Variable	All crashes		Crashes with driver performance- associated factors^*a*^	
	Total number	Daily average ± SD	Total number (%)	Daily average ± SD
Year
2000	9,956	64.6 ± 12.3	3,030 (30.4)	19.7 ± 7.5
2001	9,662	62.7 ± 12.7	1,948 (20.2)	12.6 ± 4.2
2002	9,285	60.3 ± 12.4	1,462 (15.7)	9.5 ± 3.1
2003	8,802	57.2 ± 12.2	2,692 (30.6)	17.5 ± 5.1
2004	8,552	55.5 ± 11.8	2,548 (29.8)	16.5 ± 4.8
2005	9,187	59.7 ± 13.1	2,088 (22.7)	13.6 ± 4.1
2006	10,409	67.6 ± 14.4	3,553 (34.1)	23.1 ± 6.0
2007	11,201	72.7 ± 14.8	4,134 (36.9)	26.8 ± 6.3
2008	10,470	68.0 ± 14.6	4,126 (39.4)	26.8 ± 6.4
2009	10,685	69.4 ± 14.7	4,230 (39.6)	27.5 ± 6.5
2010	10,388	67.5 ± 13.5	4,196 (40.4)	27.2 ± 6.1
2011	9,892	64.2 ± 14.1	3,958 (40.0)	25.7 ± 7.0
Month
May (15th to 31st)	13,864	68.0 ± 12.4	4,422 (31.9)	20.0 ± 10.1
June	25,174	69.9 ± 13.9	8,045 (32.0)	20.6 ± 10.1
July	26,092	70.1 ± 13.1	8,284 (31.7)	20.6 ± 9.9
August	20,047	53.9 ± 11.8	6,716 (33.5)	16.7 ± 8.7
September	21,954	61.0 ± 12.5	6,920 (31.5)	17.7 ± 9.4
October (1st to 15th)	11,358	63.1 ± 13.7	3,578 (31.5)	18.3 ± 10.2
Day of Week
Sunday	13,664	51.8 ± 10.1	4,589 (33.6)	16.0 ± 7.9
Monday	17,279	65.5 ± 12.9	5,537 (32.0)	19.4 ± 9.9
Tuesday	17,337	65.7 ± 13.4	5,521 (31.8)	19.3 ± 10.3
Wednesday	17,394	65.9 ± 13.1	5,441 (31.3)	19.0 ± 9.7
Thursday	17,954	68.0 ± 13.6	5,706 (31.8)	20.0 ± 10.3
Friday	19,147	72.5 ± 15.3	5,904 (30.8)	20.6 ± 10.4
Saturday	15,714	59.5 ± 11.3	5,267 (33.5)	18.4 ± 9.2
Holiday
No	115,042	64.7 ± 14.0	36,822 (32.0)	19.1 ± 9.8
Yes	3,447	48.5 ± 12.9	1,143 (33.2)	14.8 ± 8.6
Beginning or end of holidays^*b*^
No	112,468	64.5 ± 14.1	35,995 (32.0)	19.0 ± 9.9
Yes	6,021	57.3 ± 15.2	1,970 (32.7)	17.9 ± 8.5
^***a***^Crashes that included among the list of concurrent factors at least one of the following: “distraction,” “driver error,” or “disease, fatigue, or sleepiness.” ^***b***^Previous day, first day, or last day of a holiday period.				

**Table 2:** Descriptive statistics on the daily number of motor vehicle crashes, temperature, and heat wave periods by climatic zone.

Climatic zone^*a*^	Population (year 2001)	Daily number of motor vehicle crashes			Weather variables				
		All crashes (Mean ± SD)	With driver performance–associated factors^*b*^		Average maximum temperature (Mean ± SD)	Average minimum temperature (Mean ± SD)	Total number of heat wave days^*c*^		Rainy days^*d*^* n *(%)
			Mean ± SD	Percent of all crashes			Based on tmax	Based on tmin	
1	354,410	3.2 ± 1.9	1.5 ± 1.3	46.7	27.9 ± 4.3	14.8 ± 3.5	163	203	912 (49)
2	241,406	2.2 ± 1.6	0.9 ± 1.0	38.1	27.2 ± 4.2	13.8 ± 3.2	92	116	975 (53)
3	130,461	0.8 ± 0.9	0.3 ± 0.6	36.8	26.1 ± 4.5	13.1 ± 3.1	67	103	840 (45)
4	280,706	3.5 ± 2.2	1.5 ± 1.6	42.5	28.1 ± 4.0	15.9 ± 3.0	197	126	687 (37)
5	175,592	2.1 ± 1.7	0.8 ± 1.0	39.8	26.5 ± 3.5	16.2 ± 2.9	184	156	806 (44)
6	3,083,672	36.9 ± 11.2	9.7 ± 5.1	26.3	25.9 ± 3.1	18.0 ± 2.9	292	263	625 (34)
7	555,969	5.6 ± 2.6	2.1 ± 1.7	37.4	27.7 ± 3.1	18.2 ± 2.9	204	209	897 (48)
8	1,300,082	7.4 ± 3.8	2.9 ± 2.4	38.6	26.5 ± 4.0	16.1 ± 3.1	147	162	723 (39)
9	62,190	0.7 ± 0.9	0.3 ± 0.5	38.2	26.8 ± 4.2	14.9 ± 3.3	218	164	817 (44)
10	83,843	0.8 ± 0.9	0.3 ± 0.6	39.0	25.8 ± 4.5	12.1 ± 3.2	71	92	797 (43)
11	11,879	0.1 ± 0.3	0.04 ± 0.2	40.0	19.8 ± 4.3	9.8 ± 3.4	43	74	885 (48)
12	47,448	0.6 ± 0.8	0.2 ± 0.5	36.6	23.7 ± 6.0	9.2 ± 3.6	21	27	687 (37)
13	7,707	0.2 ± 0.4	0.05 ± 0.2	31.3	21.7 ± 4.8	7.6 ± 3.2	71	61	542 (30)
14	7,691	0.1 ± 0.3	0.04 ± 0.2	44.4	23.4 ± 5.5	10.1 ± 3.4	27	34	747 (41)
^***a***^Climatic zones are shown in Figure 1. ^***b***^Crashes that included among the list of concurrent factors at least one of the following: “distraction,” “driver error,” or “disease, fatigue, or sleepiness.” ^***c***^Heat waves were defined as ≥ 2 consecutive days with maximum (tmax) or minimum (tmin) temperature exceeding the weather station–specific historic 95th percentile. A day in a climatic region was considered a heat wave day if at least one station in the region registered a heat wave during that day. ^***d***^A specific day in a climatic region was considered as having had some rain if at least one station in the region registered precipitation values > 0 mm.									

**Figure 2:**
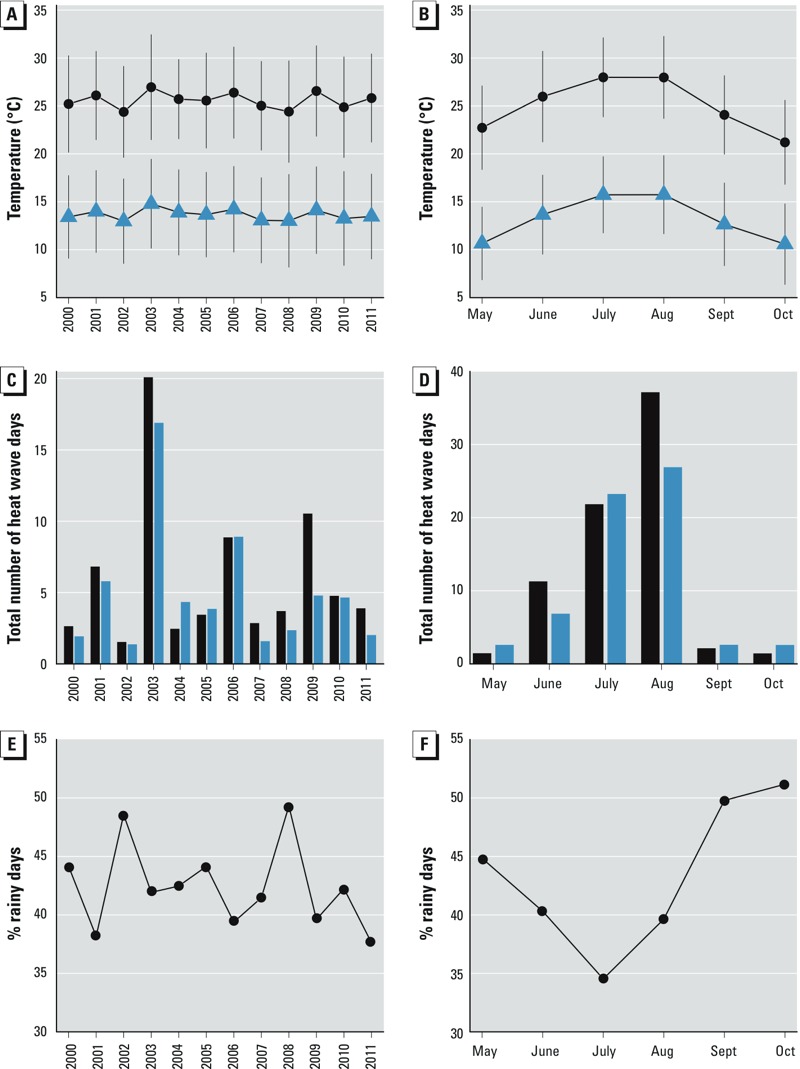
Distribution of temperatures and number of heat wave days by year and month. Black color and circles indicate maximum temperature and heat wave days defined using maximum temperature. Blue color and triangles indicate minimum temperature and heat wave days defined using minimum temperature. Panels *A* and *B* show averages ± standard deviations. Panels *C* and *D* show total number of heat wave days averaged over the 14 climatic regions of Catalonia. Panels *E* and *F* show the overall percentage of rainy days.

Average maximum temperatures did not show a trend over the years during the study period, although there was a difference of 2.5°C between the coldest and warmest years (Figure 2). A monthly pattern was observed, with July and August being the hottest months. The number of heat wave days varied substantially over the years, with 2003 and 2009 having the most heat wave days. The average maximum temperature in the different climatic regions ranged from 19.8°C to 28.1°C, and the number of heat wave days showed large variations among regions, with up to 10-fold differences (Table 2).

**Figure 3:**
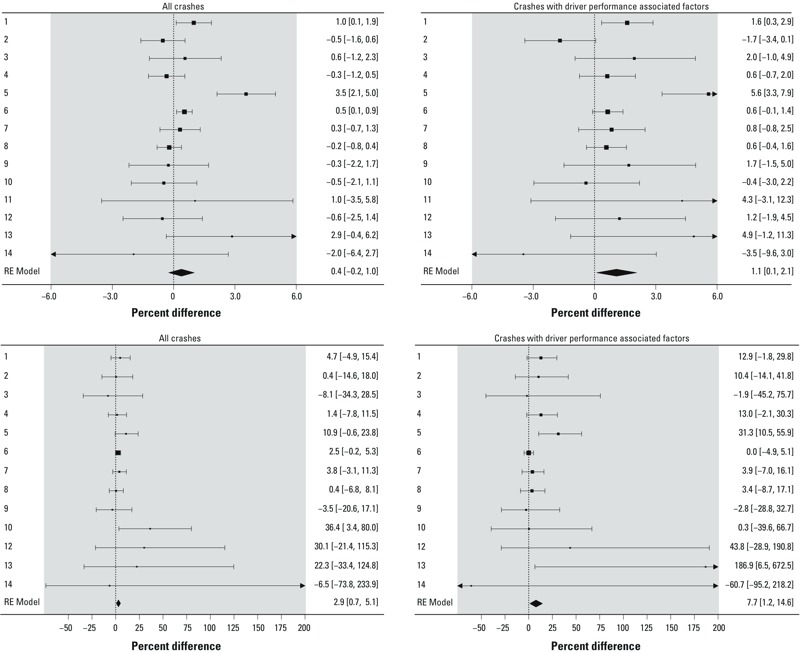
Forest plot of the associations by climatic region. The top row shows the percent difference in risk of crashes (with 95% CIs) associated with a 1°C increase in daily maximum temperature. The bottom row shows the percent difference in risk of crashes (with 95% CIs) when comparing heat wave days (defined as days belonging to a period of ≥ 2 consecutive days with maximum temperature above the historic 95th percentile) with normal days. Climatic regions are shown in Figure 1. Overall percent differences obtained from a random effects (RE) meta-analysis model.

The risk of crashes significantly increased by 2.9% (95% CI: 0.7, 5.1%) during heat wave days, and this association was stronger (7.7%, 95% CI: 1.2, 14.6%) when the analyses were restricted to crashes with driver performance–associated factors (Table 3). The risk of these crashes significantly increased by 1.1% (95% CI: 0.1, 2.1%) for each 1°C increase in maximum temperature, although the total number of crashes was not significantly associated with maximum temperature. No significant associations with minimum temperature were found. When the study period was split into 4-year periods, the associations were stronger in the last period, that is, 2008–2011, except for the association of heat wave days with crashes involving driver performance–associated factors (Table 4). Figure 3 displays the climatic region–specific risk estimates. The associations for crashes with driver performance–associated factors were higher or less negative than those for total crashes in most regions. Heterogeneity of associations by region was higher when using maximum temperature (*I*^2^ values were 75% and 72% for all crashes and for crashes with driver performance–associated factors, respectively) than when using heat wave days (*I*^2^ values were 0% and 31%, respectively).

**Table 3:** Association between temperature and daily number of motor vehicle crashes.

Weather variable	All crashes		Crashes with driver performance–associated factors^*a*^	
	Percent difference^*b*^ (95% CI)	*p*-Value	Percent difference^*b*^ (95% CI)	*p*-Value
Maximum temperature (°C)	0.4 (–0.2, 1.0)	0.232	1.1 (0.1, 2.1)	0.029
Heat wave day (tmax)^*c*^ (yes/no)	2.9 (0.7, 5.1)	0.009	7.7 (1.2, 14.6)	0.019
Minimum temperature (°C)	0.4 (–0.1, 0.9)	0.156	0.6 (–0.4, 1.7)	0.252
Heat wave day (tmin)^*d*^ (yes/no)	–0.2 (–3.4, 3.2)	0.913	3.1 (–2.1, 8.6)	0.253
^***a***^Crashes that included among the list of concurrent factors at least one of the following: “distraction,” “driver error,” or “disease, fatigue, or sleepiness.” ^***b***^Percent difference in risk of crashes (with 95% confidence intervals) obtained from a meta-analysis of climatic zone-specific results. Models were adjusted for precipitation, day of the week, holidays, days at the beginning or end of a holiday period, and a strata variable uniquely identifying all combinations of year and month. Models for crashes with driver performance–associated factors were further adjusted for the daily number of motor vehicle crashes with factors unrelated to driver performance. ^***c***^Heat waves were defined as ≥ 2 consecutive days with maximum temperature exceeding the weather station–specific historic 95th percentile. ^***d***^Heat waves were defined as ≥ 2 consecutive days with minimum temperature exceeding the weather station–specific historic 95th percentile.				

**Table 4:** Association between temperature and daily number of motor vehicle crashes stratified by period.

Strata	All crashes		Crashes with driver performance–associated factors^*a*^	
	Percent difference^*b*^ (95% CI)	*p*-Value	Percent difference^*b*^ (95% CI)	*p*-Value
2000–2003
Maximum temperature (°C)	–0.1 (–1.1, 0.9)	0.803	0.4 (–0.5, 1.3)	0.337
Heat wave day (tmax)^*c*^ (yes/no)	0.9 (–3.2, 5.1)	0.676	6.6 (–4.2, 18.6)	0.241
2004–2007
Maximum temperature (°C)	0.3 (–0.7, 1.2)	0.561	0.8 (0.0, 1.6)	0.052
Heat wave day (tmax)^*c*^ (yes/no)	3.4 (–0.8, 7.8)	0.136	8.9 (1.1, 17.3)	0.025
2008–2011
Maximum temperature (°C)	0.8 (0.3, 1.3)	0.005	1.6 (0.1, 3.2)	0.039
Heat wave day (tmax)^*c*^ (yes/no)	4.7 (0.8, 8.8)	0.018	7.8 (–3.4, 20.3)	0.182
^***a***^Crashes that included among the list of concurrent factors at least one of the following: “distraction,” “driver error,” or “disease, fatigue, or sleepiness.” ^***b***^Percent difference in risk of crashes (with 95% CIs) obtained from a meta-analysis of climatic zone–specific results. Models were adjusted for precipitation, day of the week, holidays, days at the beginning or end of a holiday period, and a strata variable uniquely identifying all combinations of year and month. Models for crashes with driver performance–associated factors were further adjusted for the daily number of motor vehicle crashes with factors unrelated to driver performance. ^***c***^Heat waves were defined as ≥ 2 consecutive days with maximum temperature exceeding the weather station–specific historic 95th percentile.				

**Figure 4:**
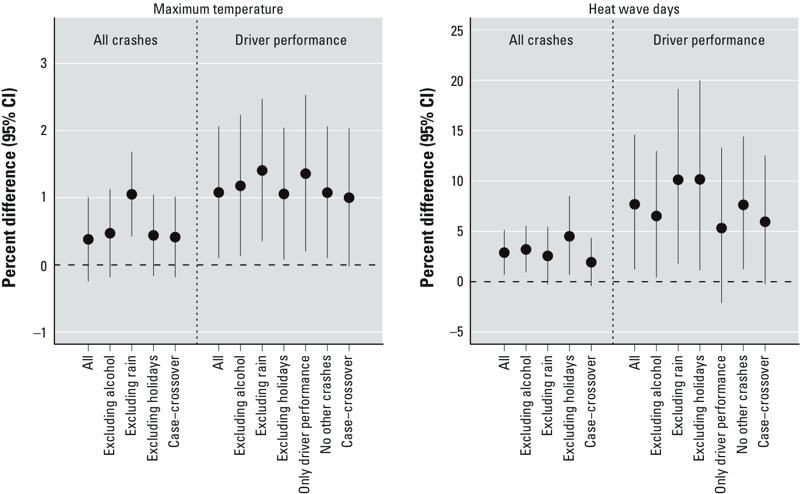
Results from sensitivity analyses. All: main results, as reported in Table 3; Case–crossover: adjusted for dummy variables based on the combination of year, month, and day of the week, resulting in a time-stratified case-crossover analysis; Excluding alcohol: crashes that had “alcohol or drugs” as concurrent factors were excluded from the analyses; Excluding holidays: holidays, Sundays, and the month of August were excluded from the analyses; Excluding rain: days with rain were excluded from the analyses; No other crashes: analyses for crashes with driver performance–associated factors were not adjusted for the number of motor vehicle crashes with factors unrelated to driver performance; Only driver performance: crashes with driver performance–associated factors as those that included “distraction,” “driver error,” or “disease, fatigue, or sleepiness” as the only concurrent factors.

The results of the sensitivity analyses produced few changes (Figure 4). Exclusion of crashes with alcohol or drugs as concurrent factors led to very similar results. In general, exclusion of days with rain or holidays, Sundays, and the month of August resulted in slightly larger estimates. The case-crossover analysis produced very similar results, although they did not reach statistical significance. Considering crashes that exclusively had driver performance–associated factors versus crashes that had driver performance–associated factors plus possible additional factors led to a reduction of 40% in the number of crashes, and the resulting RR for maximum temperature was slightly increased, whereas the RR for heat wave days was slightly decreased. Adjusting or not for crashes unrelated to driver performance did not change the results. Autocorrelation of residuals was inspected up to lag 14 (data not shown). Most of the autocorrelation values were below 0.15. Visual inspection of GAM models confirmed linearity of the associations (data not shown).

## Discussion

In this study, we estimated the effects of high temperatures and heat waves on the risk of motor vehicle crashes during the warm months in Catalonia, Spain. Overall, the risk of crashes was found to significantly increase during heat waves, especially for crashes that involved factors such as distractions, driver error, fatigue, or sleepiness. This observed pattern was consistent with our hypothesized mechanism of reduced driving performance in hot conditions. In particular, the estimated risk of crashes associated with driver performance was 7.7% higher (95% CI: 1.2, 14.6%) during heat wave periods when compared with similar warm season days not affected by extreme heat. In addition, we consistently found that the estimated risk of these crashes increased continuously with the maximum temperature of the day during the warm season, with a 1.1% increase (95% CI: 0.1, 2.1%) in risk of crashes for each 1°C increase in maximum temperature. No significant associations were found when using the minimum temperature.

Several experimental studies have shown that drivers in a hot environment make more technical errors, show an increased tendency to drift out of the lane, make more large steering adjustments, miss more signals, report more fatigue, and have lower overall driving performance (Daanen et al. 2003; Mackie and O’Hanlon 1977; Walker et al. 2001; Wyon et al. 1996). Heat has also been shown to decrease the performance of physical and intellectual tasks (Ramsey 1995; Ramsey et al. 1983), which has been linked to increases in falls and injuries (Basagaña 2014; Basagaña et al. 2011; Xiang et al. 2014). The categories included in our definition of driver performance–associated factors are likely to cover the symptoms just described, as they include distractions from the sustained attention needed for driving, as well as driver errors and fatigue. These categories, however, are not specific to factors potentially affected by heat, as they may include, for example, distractions such as mobile phone use. In our study population, the number of crashes related to driving performance increased in association with rising temperatures during the warm season. Although other studies have reported that falling asleep while driving is more common in summer than in winter (Radun and Radun 2006), to our knowledge, the present study is the first one to use daily data to examine the relationship between temperature and motor vehicle crashes linked to distractions, driver error, fatigue, or sleepiness.

We used a time series of daily data from over a decade to assess the association between temperature and number of crashes. Some relationships can be masked when using wider aggregations such as monthly data (Eisenberg 2004). Only a few previous studies have used daily data, and all of them included a substantially smaller number of crashes than our study. A study of three cities in the Netherlands estimated that more crashes were expected to occur when the temperature exceeded the monthly average temperature (Brijs et al. 2008). A study in the Balearic Islands (Spain) found no association between average temperature and the number of crashes (Rosselló and Saenz-de-Miera 2011). A study in the Athens region (Greece) found that the number of crashes was 5% higher on days with average temperature > 30°C than on days with temperature between 20°C and 30°C (Bergel-Hayat et al. 2013; Yannis and Karlaftis 2010), which is a higher estimate than the 2.9% increase in total crashes that we found associated to heat waves. The study in Greece was the largest of the three, with approximately 25 crashes per day, whereas our study had an average of 64.

The magnitude of the estimated effects of heat in our study population, for example, a 7.7% increase in crash risk on heat wave days, is smaller than other more established risk factors, such as use of cell phone while driving, which is associated with a 300% increase in risk (McEvoy et al. 2005; Redelmeier and Tibshirani 1997), or performing secondary tasks that require 1–2 eye glances and/or 1–2 button presses, which is associated with a 40% increase in risk (Dingus et al. 2011; Klauer et al. 2010). However, although the magnitude of the relative risks reported in this paper is relatively small, they can be translated into a sizeable number of crashes at the population level and, thus, should not be ignored.

One of the main strengths of this study is the use of factors associated with the crashes to identify those crashes that may be linked to driver performance. Focusing on this type of crash addresses more clearly the specific mechanism by which heat is expected to increase risk: namely, by decreasing driver performance. However, information on factors associated with crashes is reported by the police investigating the crash and can be affected by subjectivity. We believe that potential misclassifications in attributing crashes to driver performance are not associated with temperature, and therefore, they are not expected to introduce bias but rather to reduce statistical power to detect the association (Armstrong 1998). The percentage of crashes with driver performance–associated factors varied over the years, although it stabilized in the last 4 years of the study period—the period with better-quality information, as the new crash reporting guidelines were fully implemented. Reassuringly, the analysis restricted to the last 4 years showed the strongest results.

One disadvantage of our study is the lack of data on traffic volume, as changes in traffic volume affect the number of crashes, and these changes can have a seasonal pattern or may be related to weather variables (Bergel-Hayat et al. 2013). We attempted to control for changes in traffic volume by including dummy variables for day of the week and for the strata combining year and month, which had been shown to produce estimates of associations between meteorological parameters and traffic accidents in the Netherlands that were consistent with estimates from models adjusted for traffic volume (Brijs et al. 2008). In addition, in the analyses for crashes with driver performance–associated factors, we further adjusted our models for the remaining number of daily crashes (i.e., those without driver performance–associated factors). We believe that this adjustment may produce a better control for the potential residual association between temperature and crashes that is driven by changes in traffic volume.

Our study benefited from a relatively high spatial resolution, as analyses were conducted at the small climatic region level. This approach has the advantage of assigning the local temperature to each crash instead of the regional or national average, as is commonly done (Bergel-Hayat et al. 2013). In addition, this approach better controls the temporal and seasonal patterns occurring in each region instead of using the temporal pattern in the whole study region. Our analysis assessed the effects of temperature by comparing data on crashes within the same climatic region and within the same month and year. In addition, we controlled for day of the week, holiday periods, and precipitation. These strategies attempted to minimize the role of confounding by unobserved variables. In addition, our sensitivity analyses excluded potential confounding by alcohol or drugs.

We found a significant relationship between high temperature and the risk of crashes with driver performance–associated factors in Catalonia, where 86% of the vehicles had air conditioning in 2008 (Instituto Nacional de Estadística 2008). Some experimental studies have shown that having a comfortable temperature inside the vehicle improves driving performance (Daanen et al. 2003; Wyon et al. 1996). We could not investigate whether the associations found were mainly driven by the 14% of vehicles without air conditioning. Alternative potential explanations for the associations could be an inadequate use of air conditioning, that air conditioning is not sufficient to completely offset the reduced performance induced by heat, or that the effects of high temperature are indirect via, for example, dehydration or reduction in sleep quality on hot days (Grandjean and Grandjean 2007; Radun and Radun 2006; Watson et al. 2015). Another explanation is that extreme heat influences pavement conditions (e.g., pavement softening), which may then be related to the risk of crashes (Mills and Andrey 2002). Additional studies conducted in warmer areas and in areas with fewer cars with air conditioning can shed more light on the global implications of these results.

## Conclusion

The results of our study suggest that there is an increased risk of motor vehicle crashes involving driver performance–associated factors when maximum daily temperatures increase and specifically during heat waves. The future climate is predicted to have more frequent heat waves, which are also predicted to be more intense and longer lasting (Kirtman et al. 2013). Our findings on the impact of these episodes on the risk of motor vehicle crashes, if confirmed by future studies, can ultimately inform policy makers, health professionals, and clinicians regarding this potential effect of extreme heat episodes when incorporating such evidence into adaptation policies, recommendations, and targeted interventions.
